# Supplementary material for: IKK2/NFkB signaling controls lung resident CD8+ T cell memory during influenza infection
Source: Nat Commun. 2023 Jul 19;14:4331. doi: 10.1038/s41467-023-40107-1 (PMC10356942; doi:10.1038/s41467-023-40107-1)
Supplement: Supplementary file 1 — Supplementary Information [file 41467_2023_40107_MOESM1_ESM.pdf]

SUPPLEMENTARY METHODS

Reagents. Biotinylated, influenza-specific monomers (H-2Db NP366-374 ASNENMETM, H-2D^b PA224-233 SSLENFRAYV, I-A^b NP311-325 QVYSLIRPNENPAHK) were obtained from the NIH Tetramer Core Facility (Atlanta, GA). Biotinylated H-2K^b monomers for OVA (SIINFEKL) and VSV NP52-59 (RGYVYQGL) were generated in our laboratory. Biotinylated monomers were tetramerized using fluorescently labeled streptavidin (Biolegend, San Diego, CA). Doxycycline containing diet (6 g/kg) was purchased from Envigo (Indianapolis, IN).

Supplementary Table 1

**Antibodies and reagents**					
**Antigen**	**Clone**	**Fluorochromes**	**Catalog Numbers**	**Dilution**	**Vendor**
**CD8**	53-6.7	BV785, PE-Cy7, APC, PE	#100750, #100722, #100712, #100708	1:200	Biolegend
**CD4**	L3T4	BV785	#100552	1:200	Biolegend
**CD45.2**	104	PE, PE-Cy7	#109808, #109830	1:200	Biolegend
**CD103**	M290	APC/BV605	#562772, #740355	1:100	BD Biosciences
**CD44**	IM7	BV605	#103047	1:400	Biolegend
**CD62L**	MEL-14	BV785	#104440	1:200	Biolegend
**TNF**	MP6-XT22	BV785	#506341	1:100	Biolegend
**Anti-TNF blocking antibody**	MP6-XT22	ULTRALEAF PURIFIED	#506332	100mg per mouse	Biolegend
**Rat IgG1-k isotype control**	RTK2071	ULTRALEAF PURIFIED	#400432	100mg per mouse	Biolegend
**IFN-g**	XMG1.2	BV421	#563376	1:100	BD Biosciences
**CD69**	H1.2F3	APC-Cy7	#104526	1:200	BD Biosciences
**CD178 or FasL**	MFL3	PE	#106606	1:100	BD Biosciences
**Cleaved caspase-3**	D3E9	AF647	#9602S	1:100	Cell Signaling
**CXCR3**	CXCR3-173	BV421	#126529	1:100	Biolegend
**Runx3**	527327	APC, AF488	#IC3765A, #IC3765G	1:100	R&D Systems
**CD122**	TM-b1	PE, APC	#123210, #564762	1:100	BD Biosciences
**Blimp1**	6D3	BV421	#565276	1:100	BD Biosciences
**Tbet**	O4-46	BV786	#564141	1:100	BD Biosciences
**Eomes**	Dan11mag	AF660	#606-4875-82	1:100	Thermo Scientific
**Phospho-SMAD2/3**	O72-670	PE	#562586	1:50	BD Biosciences

**Phospho-NF-kBp65(Ser536)**	93H1	AF488, AF647	#4886S, #4887S	1:50	Cell Signaling
**Luciferase**	Polyclonal	Biotinylated/ Streptavidin-FITC	#200-106-150, #SA1001	1:50	Rockland, Inc.
**NK1.1**	PK136	PE-Cy7	#108713	1:100	BD Biosciences
**CD3e**	145-2C11	PerCP	#553067	1:100	BD Biosciences
**CD11c**	N418	FITC	#117306	1:100	BD Biosciences
**CD86**	GL1	PE	#553692	1:100	BD Biosciences
**BrdU**			#423401		Biolegend
**Anti-BrdU**	3D4	AF647	#364108	1:100	Biolegend
**Cytofix/Cytoperm kit**			#554714		BD Biosciences
**AccuStart II mouse genotyping kit**			#95135-100		VWR inc
**D-Luciferin**			LUCK-1G		GoldBio
**625 Doxycycline rodent diet**			TD.01306	(6 g/kg)	Envigo

Supplementary Table 2

**Primer name**	**Primer sequence**
rtTA forward	5'-GTGATTAACAGCGCATTA-3'
rtTA reverse	5'-ATCAATTCAAGGCCGAAT-3'
IKK2 forward	5'-GGTACCCGGGGATCCTCTAGTCAG-3'
IKK2 reverse	5'-GGTCACTGTGTACTTCTGCTGCTCCAG-3'

Supplementary Table 3

**Antibodies for Western Blotting**			
**Antigen**	**Clone**	**Host Species**	**Vendor**
**phospho-SMAD2(Ser465/Ser467)**	E8F3R	Rabbit mAb	Cell Signaling
**SMAD2/3**	D7G7	Rabbit mAb	Cell Signaling
**p-Erk1/2**		Rabbit Polyclonal	Cell Signaling
**alpha-tubulin**	B-5	Mouse mAB	Sigma Aldrich
**SMAD7**	293739	Mouse mAb	R&D Systems
**Goat anti-mouse**		Goat Polyclonal	Li-Cor
**Goat anti-rabbit**		Goat Polyclonal	Li-Cor

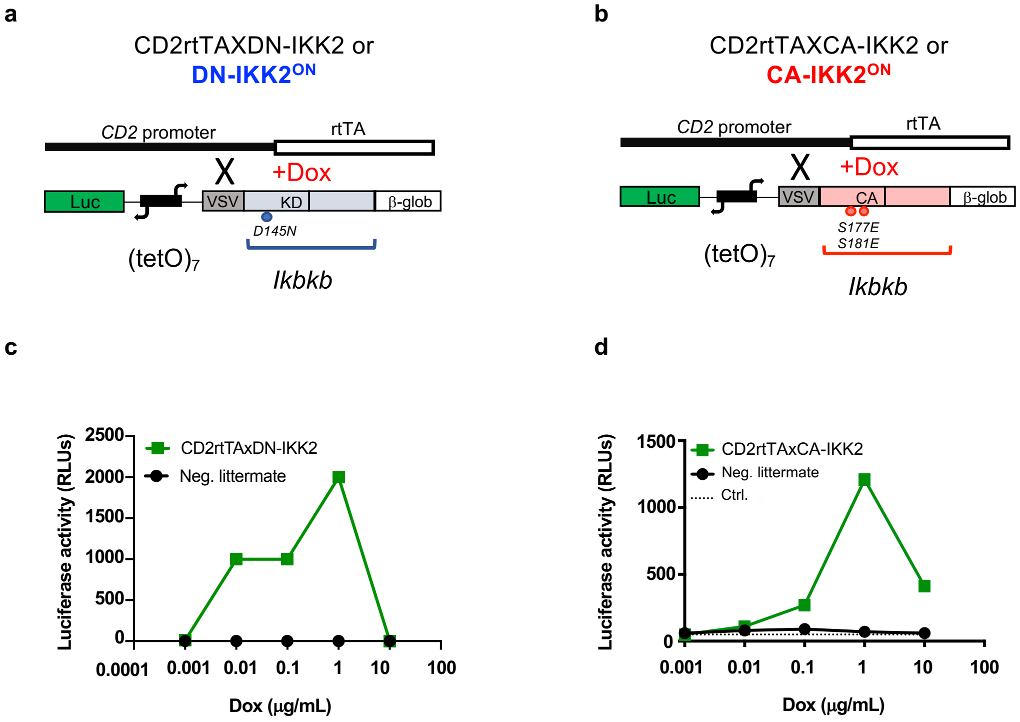

Supplementary Figure 1. T cell restricted Tet ON inducible models to inhibit or enhanced IKK2 enzymatic activity (**a-b**) Genetic strategy to generate T cell restricted inducible models DN-IKK2^{ON} (**a**) and CA-IKK2^{ON} (**b**) from crossing CD2rtTA mice generated in (Legname et al. Immunity 2000) and CA-IKK2 and DN-IKK2 described in (Herrmann et al. Nat. Med. 2005 and Sunami et al. Hepatology 2012). The tetracycline-responsive transactivator domain rtTA is constitutively expressed as a transgene in the T cell lineage driven by human CD2 regulatory regions. The rtTA-activated promoter (tetO)7 directs the transcription of luciferase and *ikbkb* that has been mutated in the kinase domain (KD, D145N; CA, S177E, S181E). VSV, vesicular stomatitis tag; β-glob, β-globin intron/poly(A) signal. In mice resulting from the cross of CD2rtTA and CA-IKK2 or DN-IKK2 strains constitutive and death kinase forms of the kinase IKK2 are expressed only in the presence of tetracycline or its derivatives (doxycycline) in cells of the T cell lineage. (**c-d**) CD2rtTAxDN-IKK2 or CD2rtTAxCAIKK2 splenocytes or their negative littermates were stimulated with anti-CD3/28 and different doses of doxycycline (DOX). Representative of two independent experiments with one mouse per condition. Luciferase activity was measured on day 2. Source data are provided as a Source Data file.

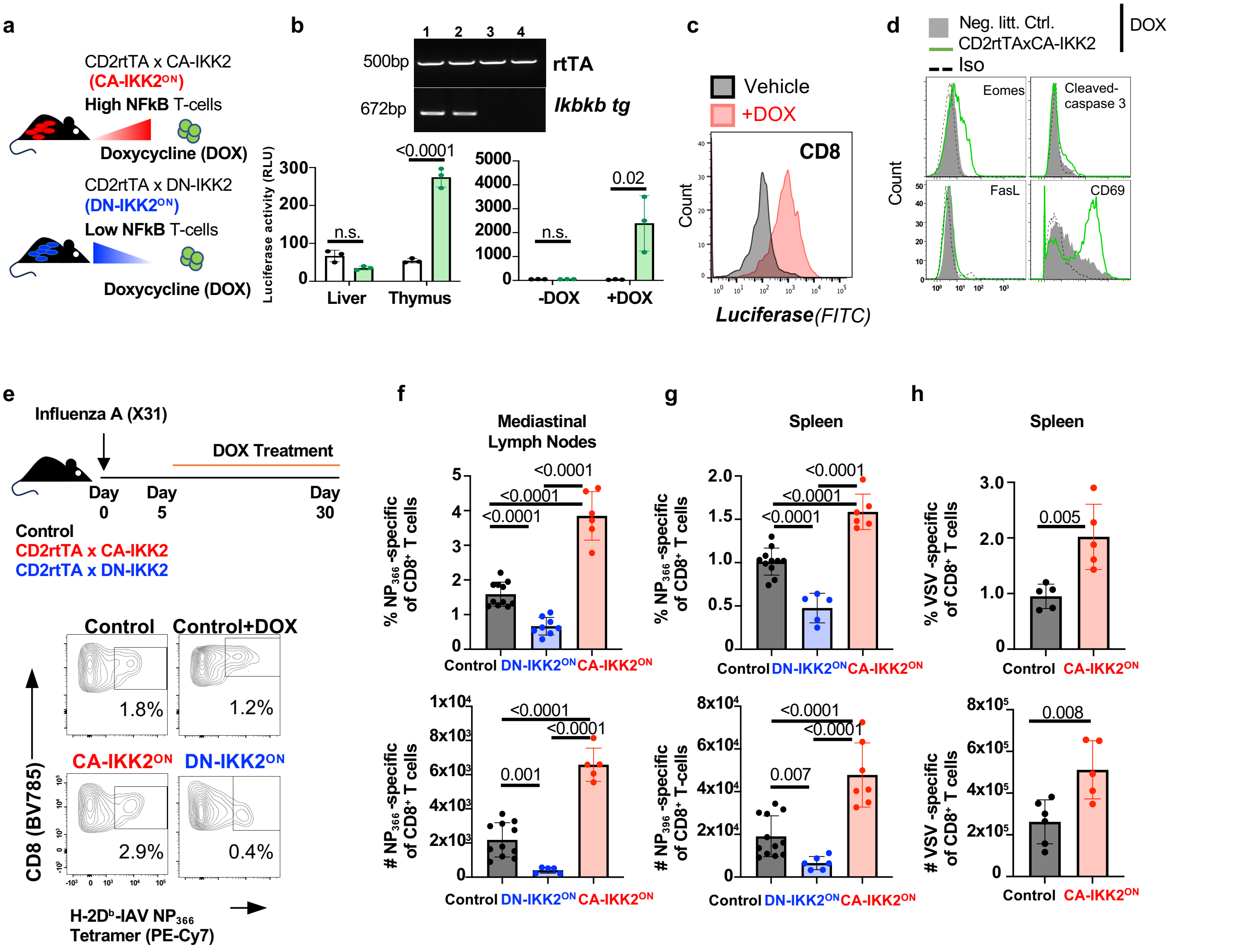

Supplementary Figure 2. Enhancing NFkB signals at the end of the immune response boosts the generation of memory CD8 T Supplementary Figure 2. (a). TetON mouse inducible models were used to enhance (CD2rtTA x CA-IKK2) or inhibit (CD2rtTA x DN-IKK2) IKK2/NFkB signaling. **(b)** PCR based analysis of indicated genes from CD2rtTA x CA-IKK2 mouse tails (lanes 1,2) and parental negative controls (lanes 3, 4). Left Graph: Liver and thymus from CD2rtTA x CA-IKK2 mice (green bars) or control littermates (green bars) treated with 4 mg/mL doxycycline solution were lysed, and luciferase activity measured to indicate absence of expression of the transgene in non-lymphoid tissues. Right graph: Purified T cells from CD2rtTAxCA-IKK2 mice (green bars) or control littler mates (white bars). were anti-CD3/CD28 stimulated with or without DOX. Luciferase activity was assessed in an enzymatic assay. n=3 mice representative of 2 independent experiments. **(c)** IKK2 constitutive activation correlates with induction of Luciferase-reporter expression measured by flow cytometry with a Luciferase-specific antibody in CD8 T cells from the lymph nodes of CD2rtTA x CA-IKK2 mice treated with DOX containing chow for 25 days. (**d**) Expression of markers indicated in CD8 T cells of CD2rtTA x CAIKK2 mice (green) or negative littermates (grey) that have been treated with DOX solution for 7 days. (**e-h**). Groups of control, CD2rtTA x CA-IKK2 (CA-IKK2^{ON}), or CD2rtTA x DN-IKK2 (DN-IKK2^{ON}) mice were infected with influenza X31 (1000 pfu). From day 5-30 post-infection (p.i.), negative littermates (control + DOX) and inducible mice were fed a doxycycline containing diet or control diet (control). Representative dot plots showing influenza NP$_{366-374}$ specific CD8^{+} T cells (Db-NP-tet^{+}, CD8^{+} CD44^{hi}) in the mediastinal lymph nodes **(e).** Frequencies and number of influenza NP specific CD8 T cells in the mediastinal lymph nodes. Combined data for %, #: n=11, 11 (control), n=8, 5 (DN-IKK2^{ON}), n=6, 5 (CA-IKK2^{ON}) mice from at least 2 independent experiments **(f).** Frequencies and number of influenza NP specific CD8 T cells in the spleen. Combined data for %, #: n=11, 12 (control), n=5, 6 (DN-IKK2^{ON}), n=6, 7 (CA-IKK2^{ON}) mice from at least 2 independent experiments **(g)** at day 30 p.i. **(h)** Groups of control or CD2rtTA x CA-IKK2 (CA-IKK2^{ON}) mice were infected with 2x10^{6} pfu vesicular stomatitis virus (VSV). Mice were fed a DOX diet from days 5 – 30 p.i. and VSV-specific memory CD8 T cells (Kb-N-tet^{+} CD8^{+} CD44^{hi}) were assessed in spleen by flow cytometry. Combined data for %, #: n=5, 6 (control), n=5, 5 (CA-IKK2^{ON}) mice pooled from 2 independent experiments**.** Bars represent mean values +/- SD. P-values were determined by two-tailed unpaired t-test, n.s. not significant. Days post infection or d.p.i. Source data are provided as a Source Data file.

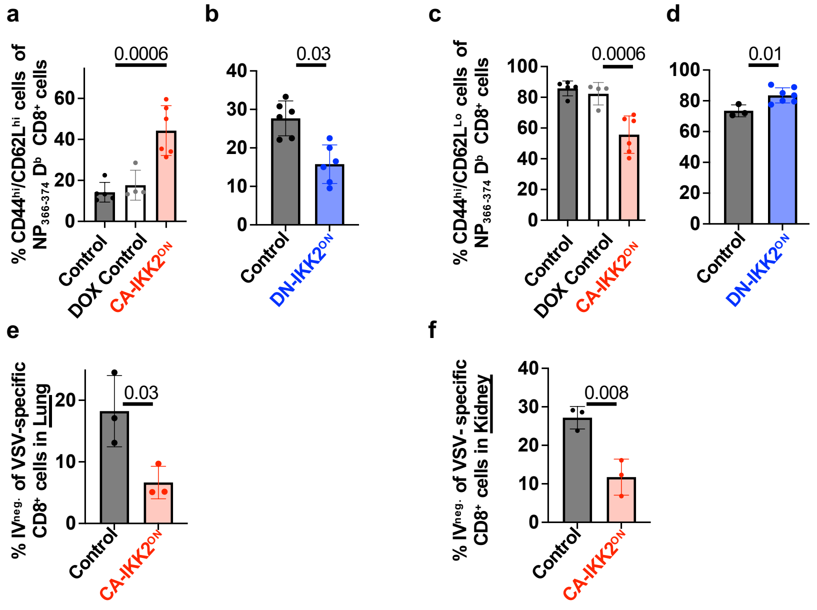

Supplementary Figure 3. Frequencies of influenza specific T_{CM} and T_{EM} in mediastinal lymph nodes (a-d) and VSV specific T_{RM} in lung and kidney (e-f). Groups of control, CD2rtTA x CA-IKK2 or CD2rtTA x DN-IKK2 mice were infected with influenza X31 (1000 pfu). From 5-30 (d.p.i.), mice were fed a DOX-containing diet (DOX control, CA-IKK2^{ON} or DN-IKK2^{ON}) or control diet (CD2rtTA x CA-IKK2; CD2rtTA x DN-IKK2 or control littermates fed with regular chow) **(a-d)**. Frequencies of influenza NP$_{366-374}$-specific CD44^{hi}CD62L^{hi} T_{MEM} (CD8$^+$ Db-NP-tet$^+$ CD44^{hi} CD62L^{hi} **(a-b)**, and CD44^{hi}CD62L^{lo} T_{MEM} (CD8$^+$ Db-NP-tet$^+$ CD44^{hi} CD62L^{lo}) (**c-d**) subsets were distinguished by flow cytometry in mediastinal lymph nodes at 30 d.p.i. n= 5 (control), n=4 (Dox control), n=6 (CA-IKK2^{ON}) in (**a,c**); n= 6 (control and DN-IKK2^{ON}) mice in (**b**); n= 3 (control), n=7 (DN-IKK2^{ON}) mice in (**d**) pooled from 3 independent experiments. **(e-f)** Groups of control or CD2rtTA x CA-IKK2 (CA-IKK2^{ON}) mice were infected with vesicular stomatitis virus (VSV). Mice were fed a DOX-containing diet from 5 – 30 d.p.i. At 30 d.p.i., VSV-specific CD8+ T_{RM} (Kb-N-tet$^+$ CD8$^+$ CD45.2$^-$) were identified in the lungs and kidneys of infected mice by IV negative staining. n=3 mice representative of 2 independent experiments. Bars represent mean values +/- SD. P-values were determined by two-tailed unpaired t-test, n.s. not significant. Days post infection or d.p.i. Related to Fig 1. Source data are provided as a Source Data file.

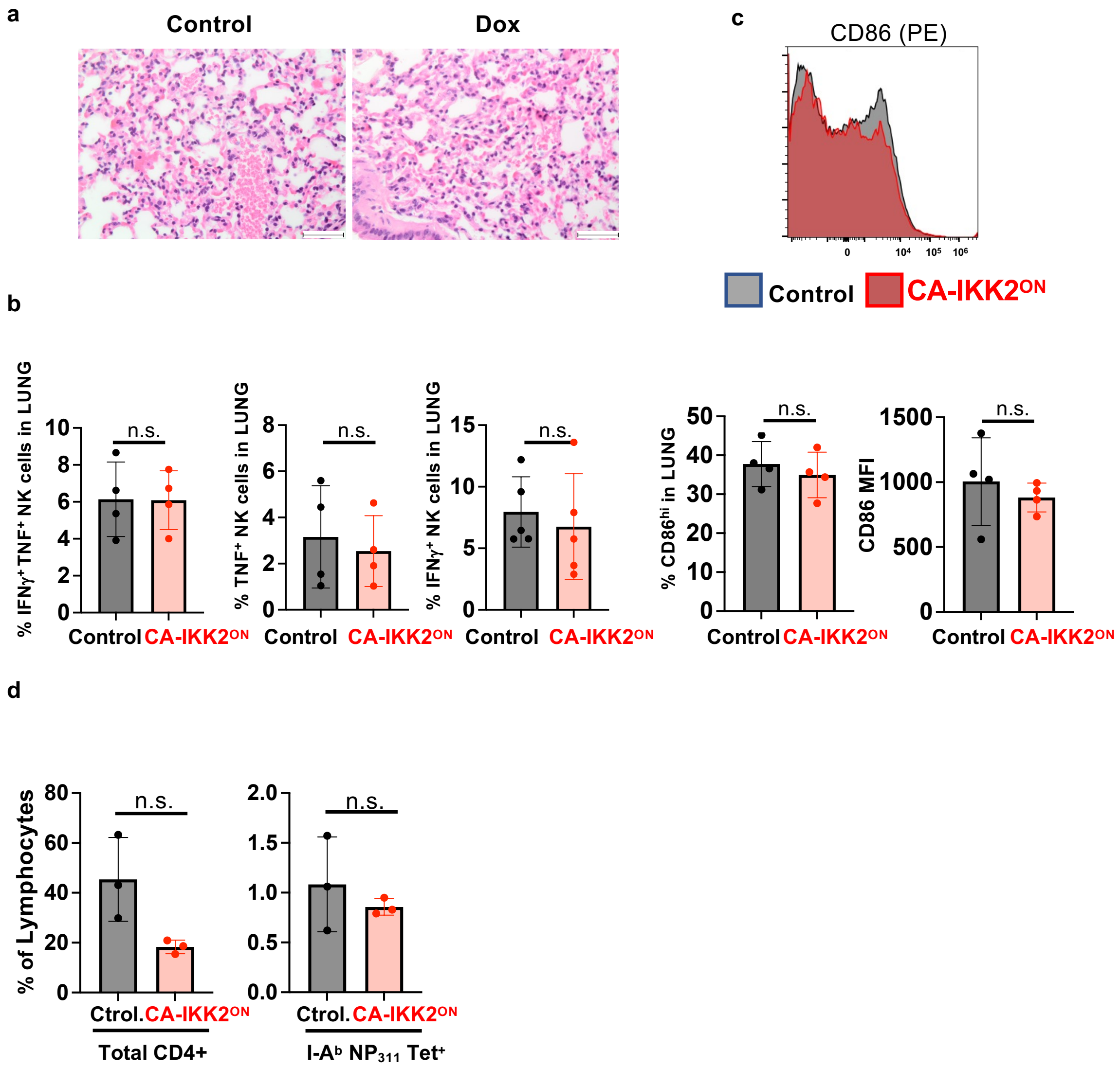

Supplementary Figure 4. a) Groups of CD2rtTA x CA-IKK2 mice were administered a DOX-containing diet or control diet. Lungs were harvested 20 d.p.i and inflammation was assessed by histopathology (H&E staining) of formalin-fixed lung sections. Analysis by the pathologist detected no discernible differences in the histopathological staining of multiple lung sections. Scale bars: 50mM. **(b and c)** Groups of CD2rtTA x CA-IKK2 mice were infected with influenza X31 (1000 pfu). A DOX-containing diet was administered to one group of mice at 5 days post infection (red bars). After 20 days, cells were isolated from the lungs of infected mice and the expression of inflammatory cytokines by natural killer cells (CD3$^-$, NK1.1$^+$ cells) was determined by intracellular staining. n=4, 5 (control), n=4 (CA-IKK2^{ON}) mice representative of 2 independent experiments(**b**). The activation state (induction of CD86) of dendritic cells (CD3$^-$ CD11c$^+$ cells) present in the lungs (**c**) was assessed by flow cytometry. n=4 mice pooled from 2 independent experiments. (**d)** Total and influenza virus NP$_{311\text{-}325}$-specific CD4 positive memory T cells were determined by flow cytometry upon influenza X31 infection (5000 pfu). n=3 mice representative of 2 independent experiments. Bars represent mean values +/- SD. P-values were determined by two-tailed unpaired t-test, n.s. not significant. Days post infection or d.p.i. Source data are provided as a Source Data file.

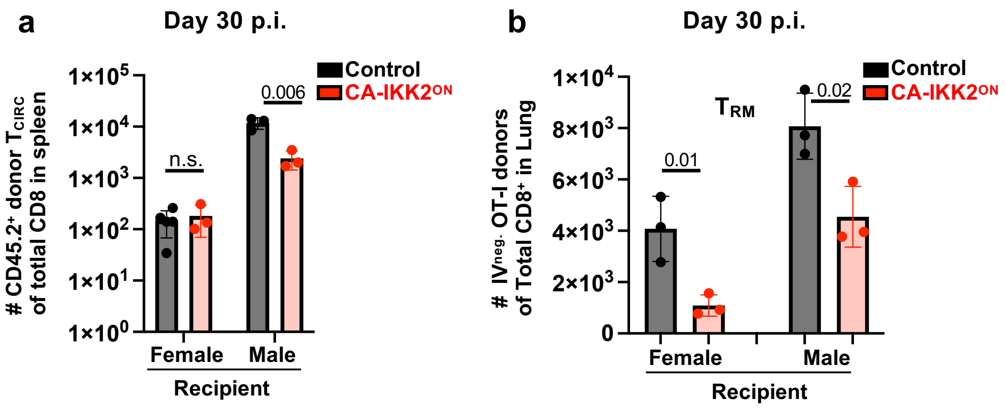

Supplementary Figure 5. Cell numbers related to Fig. 3c (a) and 3d. (a) Naive CD8 donor T cells from male OT-IxIKK2CA$^{fl/fl}$xGzB^{Cre} or OT-I littermate control mice were adoptively transferred into groups of congenically marked male and female host mice, followed by intranasal 2x10^{4} VSV-OVA infection. Graph shows number of donor CD8 T cells in the spleen at 30 d.p.i.). n=5 (female), 3 (male) control and n=3, CA-IKK2^{ON} mice representative of 2 independent experiments. **(b)** Graph shows number of lung-resident, donor OT-I CD8 T cells determined by intravascular staining in female and male hosts at 30 d.p.i. Combined data of n=3 mice representative of 2 independent experiments. Bars represent mean values +/- SD. P-values were determined by two-tailed unpaired t-test, n.s. not significant. Days post infection or d.p.i. Source data are provided as a Source Data file.

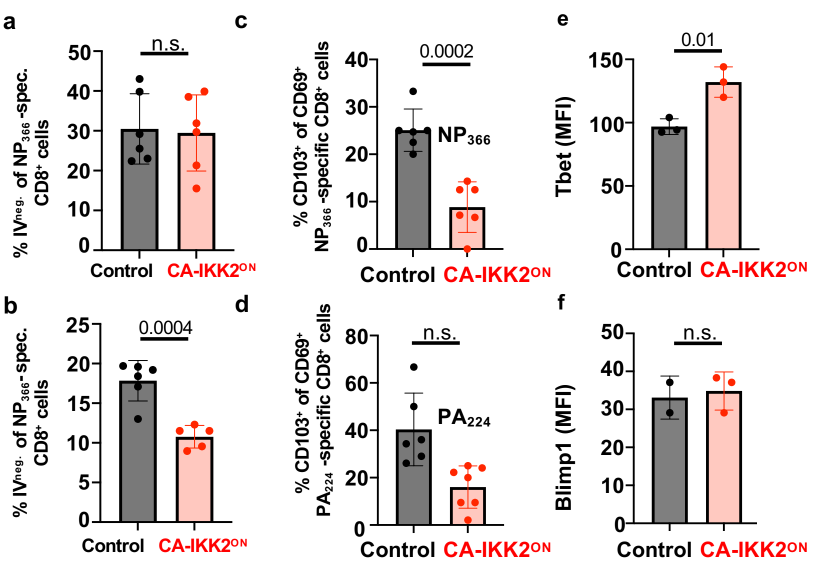

Supplementary Figure 6. Frequencies of CD103⁺ influenza specific CD8 T_{RM} cells and NF-kB-mediated regulation of memory-related transcription factors. Groups of control or CD2rtTA x CA-IKK2 (CA-IKK2^{ON}) were infected with influenza X31. Beginning at day 5 p.i., mice were fed a DOX containing diet or control diet. Influenza-specific CD8 T_{RM} were identified by intravascular staining with PE-labeled CD45.2 antibody. **(a-b)** Frequencies of CD45.2 IV^{-} cells among the $NP_{366-374}$ CD8 T_{RM} cells in the lung parenchyma at 10 (a) and 30 (b) d.p.i. Related to Fig. 4b. (**c-d**) Frequencies of CD103 positive cells among the CD69^{+} $NP_{366-374}$ specific (top graph) and $PA_{224-233}$ specific (bottom graph) CD8^{+} T_{RM} cells in the lung parenchyma at day 30 p.i. Related to Fig. 4d. (**c-d**) Expression of the transcription factors Tbet and Blimp1 was determined ex vivo in Influenza specific CD8 T_{RM} cells by flow cytometry in lung parenchyma at 30 d.p.i. Graphs in (**a-b**) show combined data of n=6 (control, CA-IKK2^{ON},**a**), n=5 (CA-IKK2^{ON}) mice in (**b**) pooled from 2 independent experiments. Graphs in (**c** ,**d**) show combined data of n=6 (control, CA-IKK2^{ON},**a**), n=7 (CA-IKK2^{ON} in (**d**)) pooled from 2 independent experiments. Graphs in (**e**) show combined data of n=3, n=2 (control (**f**)) mice representative of 2 independent experiments. Days post infection or d.p.i. Bars represent mean values +/- SD. P-values were determined by two-tailed unpaired t-test, n.s. not significant. Related to Fig 4. Source data are provided as a Source Data file.

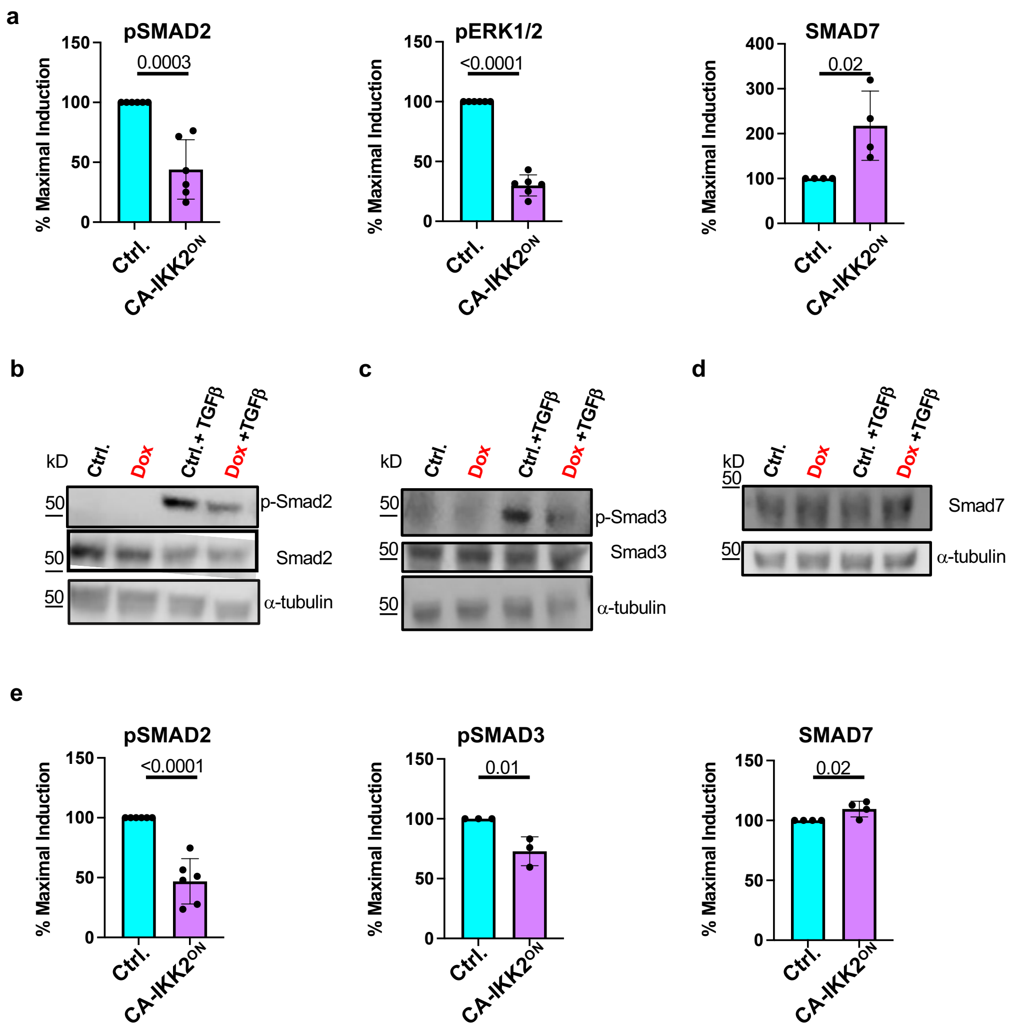

Supplementary Figure 7. NFkB inhibits TGFβ signaling in the tetON CD2rtTAxCAIKK2 inducible model. (**a**) Graphs show combined data for the percentage of maximal phosphorylated-SMAD2 (n=6), phosphorylated-ERK1/2 (n=6) and SMAD7(n=4) upon TGFβ induction in control and CA-IKK2^{ON} OT-I CD8 T cells from pooled from 3 independent experiments performed as in Fig. 5d. (**b-d**) Immunoblot and densitometry of phospho-SMAD2, -SMAD3 and SMAD7 using T cells from the CD2rtTAxCA-IKK2 tetON inducible model. DOX indicates samples where cells were treated with doxycycline to induce constitutive active IKK2 activity. Cells were stimulated as in Fig. 5d in the presence or absence of TGFβ. Samples from the same experiment run in parallel gels due to the similarity of the proteins' MW (~50kD). Blots belong to a representative experiment out of n=3 independent experiments. (**e**) Graphs show combined data for the percentage of maximal phosphorylated-SMAD2 (n=6), phosphorylated-SMAD3 (n=3) and SMAD7 (n=4) upon TGFβ induction in control and DOX inducible CA-IKK2^{ON} CD8 T cells pooled from at least 2 independent experiments. Bars represent mean values +/- SD. P-values were determined by two-tailed unpaired t-test. Related to Fig 5. Source data are provided as a Source Data file.

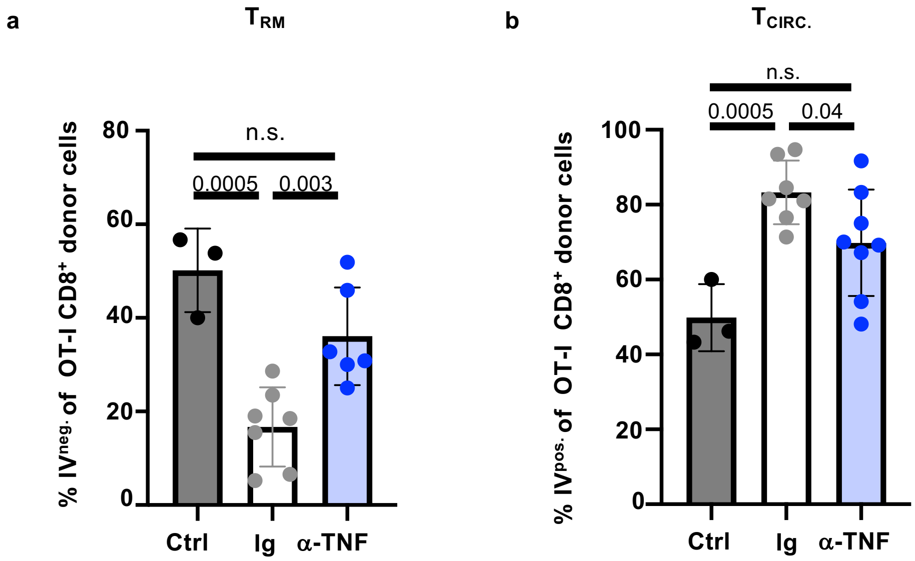

Supplementary Figure 8. TNF blockade restores T_{RM} loss caused by chronic inflammation. Frequencies of T_{RM} and T_{CIRC} upon TNF blockade. Groups of mice received $1x10^5$ congenic OT-I.CD90.1 donor CD8 T cells. Mice were infected with PR8-OVA (100 pfu). Groups were treated with either anti-TNF antibody or Rat IgG isotype control at days 10, 14, 20, 22, and 24 p.i.. Mice (except Ctrl.) were re-infected with influenza PR8 (100 pfu) at 10 d.p.i. Lung parenchyma resident cells were identified as IV negative upon injection of anti-CD8b antibody prior to euthanasia from lymphocytes isolated from the lungs at 30 d.pi. Frequency of OT-I donor resident memory CD8 ($CD8\alpha^+$, $CD90.1^+$, IV^-) (**a**) and circulating CD8 ($CD8\alpha^+$, $CD90.1^+$, IV^+) (**b**) determined in the lung by flow cytometry. Graphs in (**a-b**) show combined data from n=3 (control), 7 (Ig), 6 (a) or 8 (b) (α-TNF) mice pooled from 2 independent experiments. Days post infection or d.p.i. Bars represent mean values +/- SD. P-values were determined by two-tailed unpaired t-test, n.s. not significant. Related to Figure 5l**.** Source data are provided as a Source Data file.

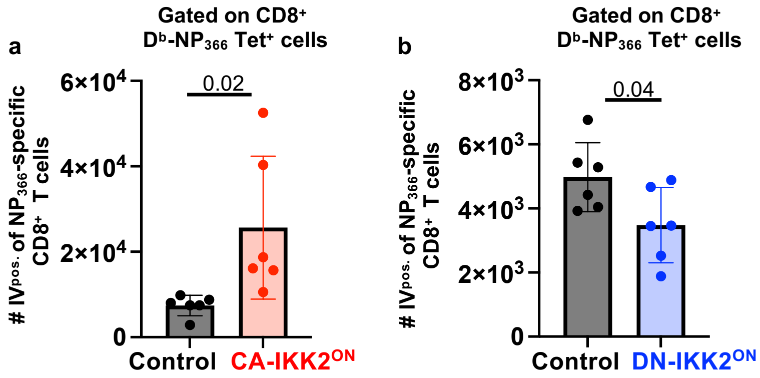

Supplementary Fig. 9. Cell numbers related to Fig 6c (a) and Fig. 6h (b). Groups of control or CD2rtTA x CA-IKK2 mice (**a**) or CD2rtTAxDN-IKK2 mice (**b**) were infected with influenza X31 (1000 pfu). At 30 d.p.i. mice were fed a DOX (CA-IKK2^{ON} or DN-IKK2^{ON}) or a control containing diet for 15 days. (**a,b**) Numbers of of circulating (IV positive), $NP_{366\text{-}374}$-specific CD8 T cells in the lung. . Graphs in (**a-b**) show combined data from n=6 mice pooled from 2 independent experiments. Bars represent mean values +/- SD. P-values were determined by two-tailed unpaired t-test, n.s. not significant. Days post infection or d.p.i. Source data are provided as a Source Data file.

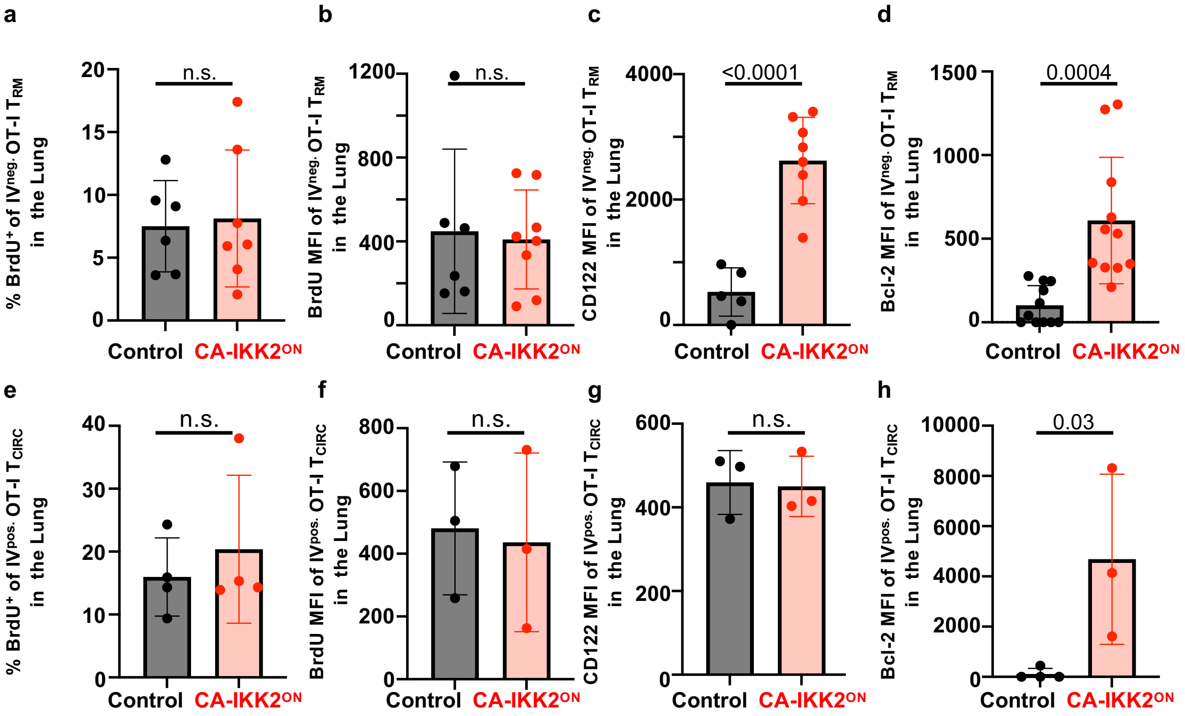

Supplementary Figure 10. Increased IKK2/NFkB signaling at memory results in a boost of lung T_{RM} maintenance that is not due to an increase in lung T_{RM} cell proliferation. Groups of congenic mice received $1x10^5$ of OT-I x CD2rtTA x CA-IKK2 CD8 T cells. Mice were infected with influenza PR8-OVA (100 pfu). At 30 d.p.i. a cohort of the mice was fed DOX or control chow. After 5 additional days, mice were given a bolus dose of BrdU (2 mg) followed by a supplemental dose (0.8 mg daily) of BrdU in their drinking water for 6 additional days. Lymphocytes were then harvested from the lungs and BrdU levels (**a-b, e-f**), along with CD122 (**c,g**) and Bcl-2 (**d,h**) expression levels were determined in resident ($CD8^+$, $CD44^+$ IV/$CD8\beta^-$, $CD90.1^+$) and circulating ($CD8^+$, $CD44^+$, IV/$CD8\beta^+$, $CD90.1^+$) donor cells by flow cytometry. Graphs show combined data from n=6 (**a**,**b**), n= 5(**c**), 11(**d**), 4(**e**), 3(**f**,**g**), 4(**h**) (control); n= 7(**a**), 8(**b**), 8(**c**), 11(**d**), 4(**e**), 3(**f,g,h**) (CA-$IKK2^{ON}$) mice pooled (**a-d**) or representative (**e-h**) from 2 independent experiments. Bars represent mean values +/- SD. P-values were determined by two-tailed unpaired t-test, n.s. not significant. Days post infection or d.p.i. Source data are provided as a Source Data file.

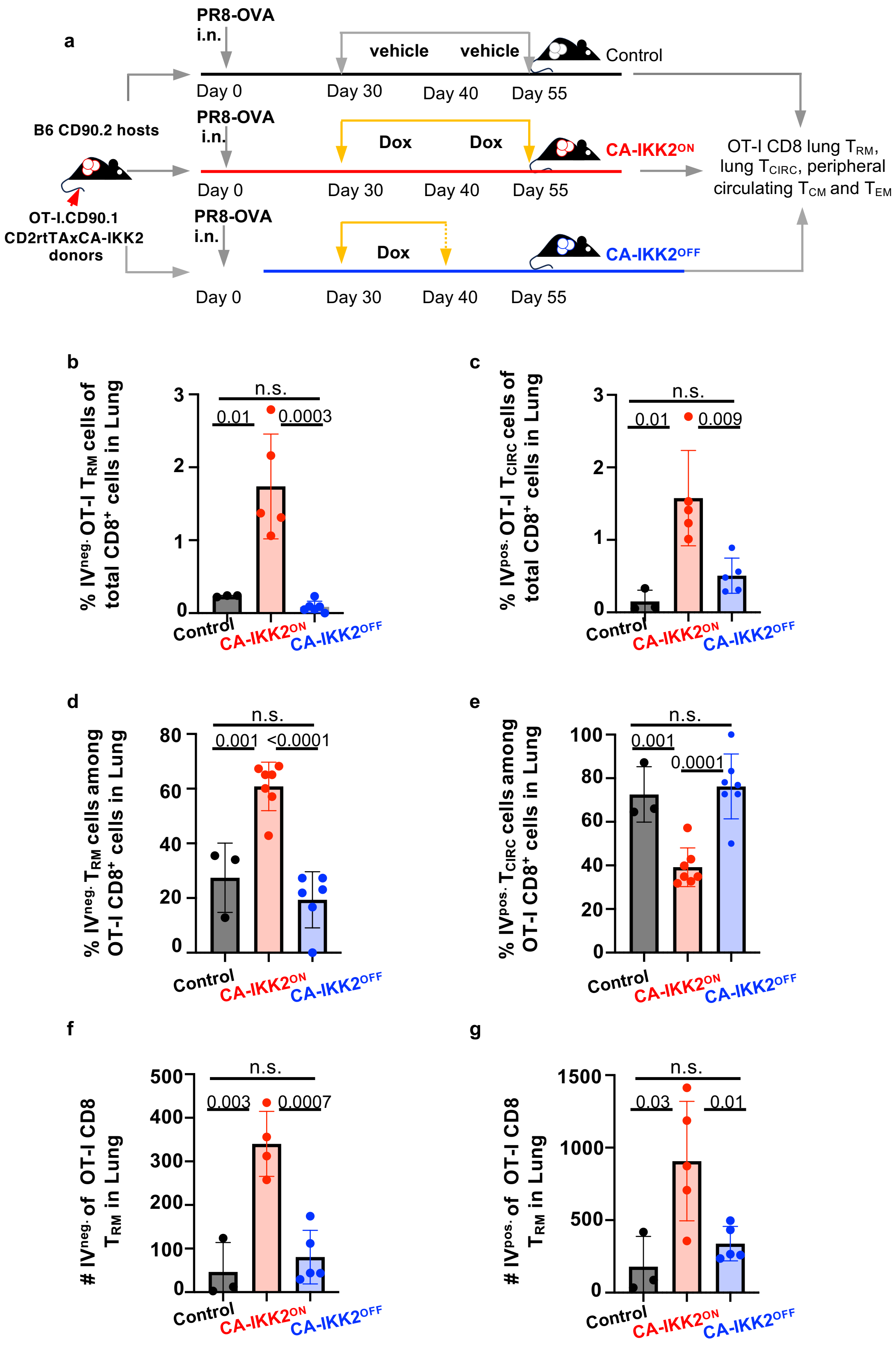

Supplementary Figure 11. Increased IKK2/NFkB signaling at memory results in a boost of lung T_{RM} maintenance that requires continuous provision of NFkB signals. (**a**) Cartoon depicting experimental design for (**b-g**). 1x10^5 OT-I.CD90.1xCD2rtTAxCA-IKK2 donor naïve CD8 T cells were adoptively transferred into B6. CD90.2 hosts followed by intranasal infection (i.n.) with influenza PR8-OVA (100 pfu). At 30 d.p.i. a cohort of mice was fed with vehicle chow (**control**) and another with DOX chow for 10 days. At day 40 p.i, DOX treatment was interrupted for half of the cohort (**CA-IKK2^{OFF}**), while the other half continued the DOX treatment (**CA-IKK2^{ON}**). At day 55 p.i, mice were euthanized and lung T_{RM} and T_{CIRC} were determined by intravital labelling. Frequencies (**b-e**) and numbers in (**f-g**) were determined as indicated in Supplementary Fig. 8 and Fig. 5l. Graph shows combined data of n=3 (control), n= 5(**b,c,g**), 7(**d,e**), 4(**f**) (CA-IKK2^{ON}), n= 6 (**b,d**), 5(**c,f**),7(**e**),5(**g**) (DN-IKK2^{ON}) mice pooled from 2 independent experiments. Bars represent mean values +/- SD. P-values were determined by two-tailed unpaired t-test, n.s. not significant. Days post infection or d.p.i. Source data are provided as a Source Data file.

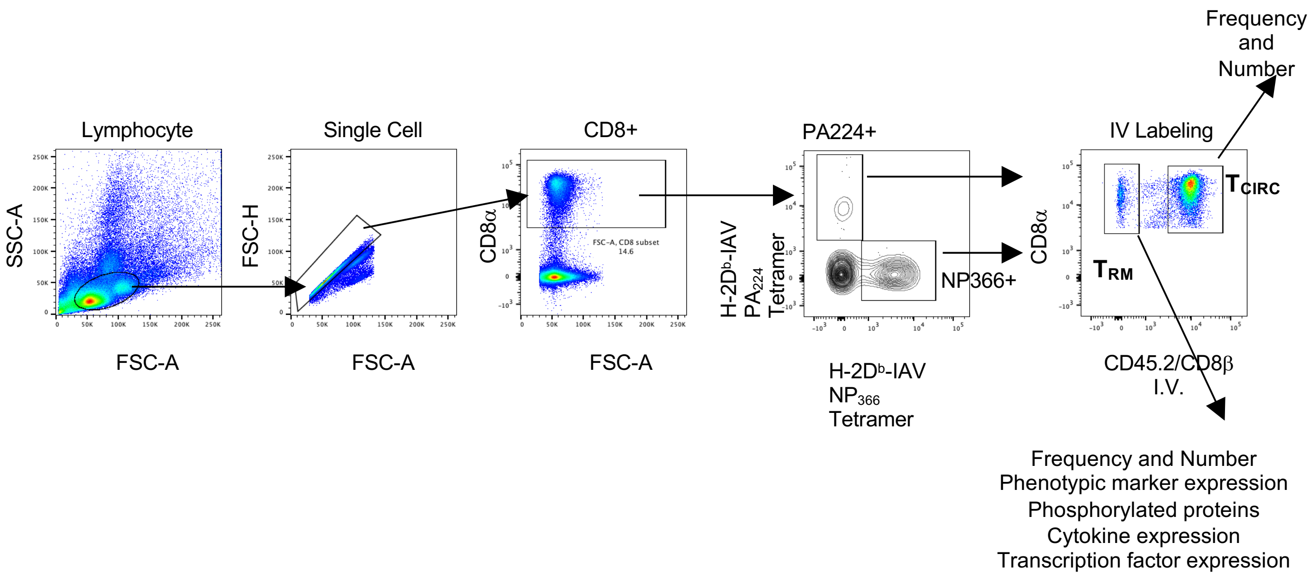

Supplementary Figure 12. Representative flow cytometry gating for the identification of circulatory (T_{CIRC}) and tissue resident (T_{RM}) influenza specific (Db-NP_{366} or Db-PA_{224} tetramer positive) $CD8^+$ T cell populations for all data included in the studies. Additionally, luciferase (Doxycycline inducible tetON models) and GFP expression (CA-IKK2 $STOP^{fl/fl}$ x Granzyme B-Cre models) positive confirmation for the expression of the CA-IKK2 or DN-IKK2 transgenes was applied (not shown in the figure). For the tetON models the $CD8^+$ T cell population is unimodal for the expression of luciferase as shown in Supplementary figure 2. Frequencies in main figures are calculated as indicated in the axis.

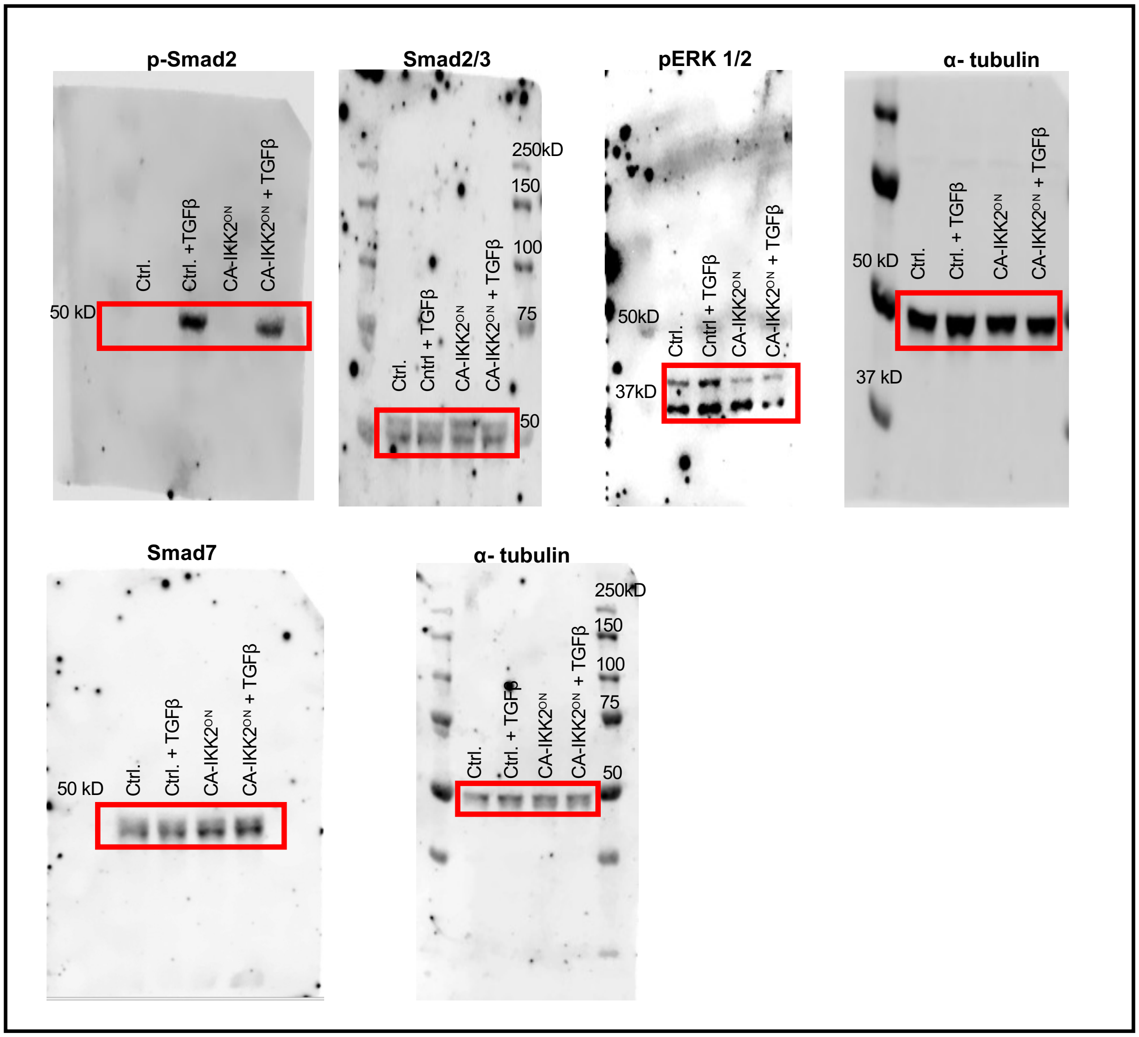

Supplementary Figure 13. Uncropped images of Western Blots shown in Fig. 5d. Red boxes show cropped regions.

Supp Figure 7b

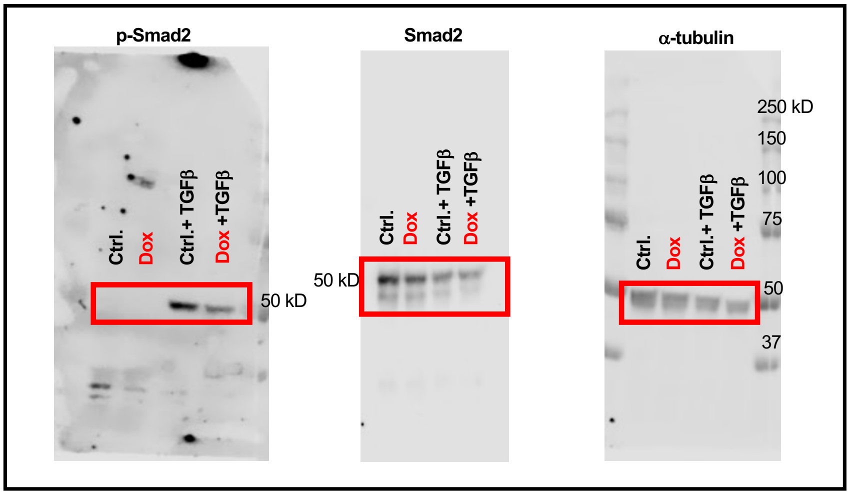

Supp Figure 7c

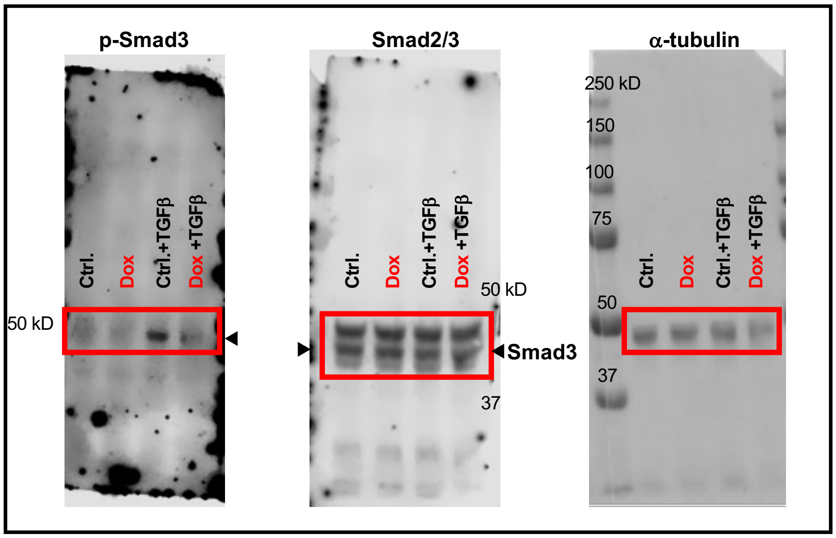

Supp Figure 7d

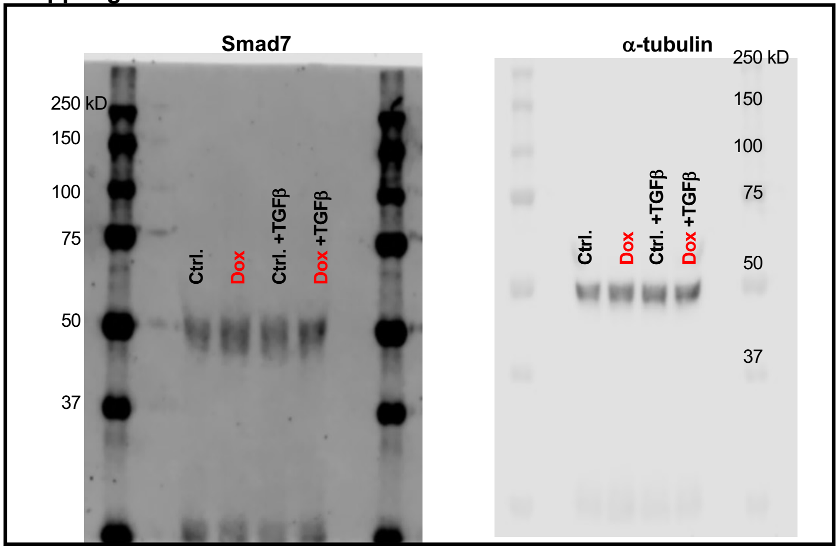

Supplementary Figure 14. Uncropped images of Western Blots shown in Supplementary Fig. 7b-d. Red boxes show cropped regions. Arrows indicate pSmad3 or Smad3 for Supplementary Fig. 7c based on MW.
