# Supplementary material for: IKK2/NFkB signaling controls lung resident CD8+ T cell memory during influenza infection
Source: Nat Commun. 2023 Jul 19;14:4331. doi: 10.1038/s41467-023-40107-1 (PMC10356942; doi:10.1038/s41467-023-40107-1)
Supplement: Supplementary file 3 — Reporting Summary [file 41467_2023_40107_MOESM3_ESM.pdf]

nature portfolio

Corresponding author(s): Emma Teixeiro

Last updated by author(s): June 24 2023

Reporting Summary

Nature Portfolio wishes to improve the reproducibility of the work that we publish. This form provides structure for consistency and transparency in reporting. For further information on Nature Portfolio policies, see our Editorial Policies and the Editorial Policy Checklist.

Statistics

For all statistical analyses, confirm that the following items are present in the figure legend, table legend, main text, or Methods section.

n/a	Confirmed	
☐	☒	The exact sample size (*n*) for each experimental group/condition, given as a discrete number and unit of measurement
☐	☒	A statement on whether measurements were taken from distinct samples or whether the same sample was measured repeatedly
☐	☒	The statistical test(s) used AND whether they are one- or two-sided<br>*Only common tests should be described solely by name; describe more complex techniques in the Methods section.*
☒	☐	A description of all covariates tested
☐	☒	A description of any assumptions or corrections, such as tests of normality and adjustment for multiple comparisons
☐	☒	A full description of the statistical parameters including central tendency (e.g. means) or other basic estimates (e.g. regression coefficient) AND variation (e.g. standard deviation) or associated estimates of uncertainty (e.g. confidence intervals)
☒	☐	For null hypothesis testing, the test statistic (e.g. *F*, *t*, *r*) with confidence intervals, effect sizes, degrees of freedom and *P* value noted<br>*Give P values as exact values whenever suitable.*
☒	☐	For Bayesian analysis, information on the choice of priors and Markov chain Monte Carlo settings
☒	☐	For hierarchical and complex designs, identification of the appropriate level for tests and full reporting of outcomes
☒	☐	Estimates of effect sizes (e.g. Cohen's *d*, Pearson's *r*), indicating how they were calculated

Our web collection on statistics for biologists contains articles on many of the points above.

Software and code

Policy information about availability of computer code

Data collection	Immunoblots were performed in Li-Cor Odyssey XF, Flow cytometry was performed on an LSRII Fortessa (BD) using FACS Diva 6.0 or an Aurora (Cytek) spectral flow cytometer using Cytek Spectraflo 3.0.3 software.
Data analysis	Image Studio Program (li-Cor v 5.2.5); FlowJo FACS analysis software (Tree Star, Inc.) version 10.4 and Graph Pad 9.4.1, Microsoft Excel 16.68.

For manuscripts utilizing custom algorithms or software that are central to the research but not yet described in published literature, software must be made available to editors and reviewers. We strongly encourage code deposition in a community repository (e.g. GitHub). See the Nature Portfolio guidelines for submitting code & software for further information.

Data

Policy information about availability of data

All manuscripts must include a data availability statement. This statement should provide the following information, where applicable:

- Accession codes, unique identifiers, or web links for publicly available datasets
- A description of any restrictions on data availability
- For clinical datasets or third party data, please ensure that the statement adheres to our policy

Raw data generated in this study have been deposited in the public Open Science Framework (OSF) https://osf.io/2vtur/, with no accession code needed. Raw flow files are available upon request due to size restrictions. Access can be obtained by contacting the corresponding author. Uncropped, unprocessed scans of the most important blots appeared in supplementary information Figures 13 and 14. The authors declare that the data supporting the findings in this study are available within the paper and its supplementary information files. Source data are provided with this article.

Research involving human participants, their data, or biological material

Policy information about studies with human participants or human data. See also policy information about sex, gender (identity/presentation), and sexual orientation and race, ethnicity and racism.

Reporting on sex and gender	N/A
Reporting on race, ethnicity, or other socially relevant groupings	N/A
Population characteristics	N/A
Recruitment	N/A
Ethics oversight	N/A

Note that full information on the approval of the study protocol must also be provided in the manuscript.

Field-specific reporting

Please select the one below that is the best fit for your research. If you are not sure, read the appropriate sections before making your selection.

[x] Life sciences   [ ] Behavioural & social sciences   [ ] Ecological, evolutionary & environmental sciences

For a reference copy of the document with all sections, see nature.com/documents/nr-reporting-summary-flat.pdf

Life sciences study design

All studies must disclose on these points even when the disclosure is negative.

Sample size	Power calculations were performed in Graph Pad Prims for multiple or two-tailed unpaired students' t-tests, using variance and effect size necessary to achieve sufficient N to determine p-values. A minimum of n=2 was chosen because it was the minimal replicate number sufficient to ascertain statistcs by t-test.
Data exclusions	No data were excluded except from data from mouse replicates where an error in the infection dose or in the staining was identified during the experimental procedure. It should be noted that the observed differences were statistically significant whether these data points were included or not.
Replication	All experiments were independently conducted a minimum or two times to ensure reproducibility. The number of biological replicates (mice) per cohort or condition for each independent experiment is noted in each Figure legend. Immunoblot analysis were repeated at least two times with completely independent biological replicates and using two different CA-IKK2ON inducible models. Biological replicates for immunoblot experiments have been noted in the pertinent figure legends.
Randomization	Mice were randomly assigned to each study group
Blinding	Blinding was performed for immunohistochemistry experiments and analysis. Blinding was not performed for other data for biosafety concerns associated with BSL2 samples.

Reporting for specific materials, systems and methods

We require information from authors about some types of materials, experimental systems and methods used in many studies. Here, indicate whether each material, system or method listed is relevant to your study. If you are not sure if a list item applies to your research, read the appropriate section before selecting a response.

Materials & experimental systems

n/a	Involved in the study	
	x	Antibodies
	x	Eukaryotic cell lines
x		Palaeontology and archaeology
	x	Animals and other organisms
x		Clinical data
x		Dual use research of concern
x		Plants

Methods

n/a	Involved in the study	
x		ChIP-seq
	x	Flow cytometry
x		MRI-based neuroimaging

Antibodies

Antibodies used

Antibodies for flow cytometry

Antigen Clone Fluorochromes Catalog Numbers Dilution Vendor
CD8 53-6.7 BV785, PE-Cy7, APC, PE #100750, #100722, #100712, #100708 1:200 Biolegend
CD4 L3T4 BV785 #100552 1:200 Biolegend
CD45.2 104 PE, PE-Cy7 #109808, #109830 1:200 Biolegend
CD103 M290 APC/BV605 #562772, #740355 1:100 BD Biosciences
CD44 IM7 BV605 #103047 1:400 Biolegend
CD62L MEL-14 BV785 #104440 1:200 Biolegend
TNF MP6-XT22 BV785 #506341 1:100 Biolegend
Anti-TNF blocking antibody MP6-XT22 ULTRALEAF PURIFIED #506332 100mg per mouse Biolegend
Rat IgG1-k isotype control RTK2071 ULTRALEAF PURIFIED #400432 100mg per mouse Biolegend
IFN-g XMG1.2 BV421 #563376 1:100 BD Biosciences
CD69 H1.2F3 APC-Cy7 #104526 1:200 BD Biosciences
CD178 or FasL MFL3 PE #106606 1:100 BD Biosciences
Cleaved caspase-3 D3E9 AF647 #9602S 1:100 Cell Signaling
CXCR3 CXCR3-173 BV421 #126529 1:100 Biolegend
Runx3 527327 APC, AF488 #IC3765A, #IC3765G 1:100 R&D Systems
CD122 TM-b1 PE, APC #123210, #564762 1:100 BD Biosciences
Blimp1 6D3 BV421 #565276 1:100 BD Biosciences
Tbet O4-46 BV786 #564141 1:100 BD Biosciences
Eomes Dan11mag AF660 #606-4875-82 1:100 Thermo Scientific
Phospho-SMAD2/3 O72-670 PE #562586 1:50 BD Biosciences
Phospho-NF-kBp65(Ser536) 93H1 AF488, AF647 #4886S, #4887S 1:50 Cell Signaling
Luciferase Polyclonal Biotinylated/ Streptavidin-FITC #200-106-150, #SA1001 1:50 Rockland, Inc.
NK1.1 PK136 PE-Cy7 #108713 1:100 BD Biosciences
CD3e 145-2C11 PerCP #553067 1:100 BD Biosciences
CD11c N418 FITC #117306 1:100 BD Biosciences
CD86 GL1 PE #553692 1:100 BD Biosciences
BrdU #423401 Biolegend
Anti-BrdU 3D4 AF647 #364108 1:100 Biolegend

Antibodies for Western Blotting
Antigen Clone Host Species Vendor
phospho-SMAD2(Ser465/Ser467) E8F3R Rabbit mAb Cell Signaling
SMAD2/3 D7G7 Rabbit mAb Cell Signaling
p-Erk1/2 Rabbit Polyclonal Cell Signaling
alpha-tubulin B-5 Mouse mAB Sigma Aldrich
SMAD7 293739 Mouse mAb R&D Systems
Goat anti-mouse Goat Polyclonal Li-Cor
Goat anti-rabbit Goat Polyclonal Li-Cor

Validation

All antibodies were validated by the manufacturers following consistent performance and specific binding activity (BD, Biolegend, Rockland, Cell Signaling, R&D, ThermoScientific and Sigma). Details can be found in the manufacturer's websites by referring to the catalague number of the antibodies above. In addition, specific detection was confirmed by us: (1) by ensuring lack of signal with isotype controls conjugated with same fluorochrome or (2) by lack of signal upon staining of the specific antibody in control populations that do not express the antigen. Please see Fig. 5b,c,e. Supplemental Fig. 2d.

Eukaryotic cell lines

Policy information about cell lines and Sex and Gender in Research

Cell line source(s)	MDCK from ATCC.
Authentication	Authentication was assessed by morphology for a epithelial cell phenotype
Mycoplasma contamination	These cells were mycoplasma negative
Commonly misidentified lines (See ICLAC register)	No misidentified cell line was used in the study.

Animals and other research organisms

Policy information about studies involving animals; ARRIVE guidelines recommended for reporting animal research, and Sex and Gender in Research

Laboratory animals	OT-1Thy1.1+ TCR transgenic strain, C57BL/6J (B6), B6.SJL-Ptprca Pepcb/BoyJ (CD45.1 congenic C57BL/6), B6.Cg-Gt(ROSA)26Sortm4 (Ikbkb)Rsky/J (IKK2-CAfl/fl), B6 -Tg (GzB-cre)1Jcb/J (GzB-Cre) mice (Jackson Laboratory, Bar Harbor, ME) along with OT-1Thy1.1XIKK2CAfl/fl xGzBCre ; CD2rtTA x CA-IKK2 (tetracycline-inducible constitutive active IKK2) and CD2rtTA x DN-IKK2 (tetracycline-inducible dominant negative IKK2) mice were maintained under specific pathogen-free conditions at the University of Missouri. All mouse strains are on the C57BL/6 background and were screened for transgene homozigocity by PCR. Mice were aged between 8-13 weeks at the time of infection for all experiments. Female and male mice were not disaggregated as no gender bias was found. Experiments in Fig. 3 required designated genders otherwise, experiments were gender matched. Infection and maintenance of mice infected with influenza virus or vesicular stomatitis virus occurred in an ABSL2 facility at the University of Missouri. Mice were euthanized by CO2 inhalation followed by cervical dislocation. Mouse housing conditions: Light/dark cycle: 12/12 with lights on at 7 AM and off at 7 PM. Ambient temperature: 70-71oF. Humidity: 50-55%. All animal procedures were conducted according to the NIH guidelines for the care and use of laboratory animals and were approved by the University of Missouri Institutional Animal Care and use Committee.
Wild animals	NO wild animals were used.
Reporting on sex	Female and male mice were not disaggregated as no gender bias was found. Experiments in Fig. 3 required designated genders otherwise, experiments were gender matched following similar studies using the same approach that were published in the peer reviewed Journal Nature 2011 (specific citation reference in main manuscript).
Field-collected samples	N/A
Ethics oversight	All procedures were conducted according to the NIH guidelines for the care and use of laboratory animals and biological safety and were approved by the University of Missouri Institutional Animal Care and use and Institutional Biosafety Committees.

Note that full information on the approval of the study protocol must also be provided in the manuscript.

Flow Cytometry

Plots

Confirm that:

- [x] The axis labels state the marker and fluorochrome used (e.g. CD4-FITC).
- [x] The axis scales are clearly visible. Include numbers along axes only for bottom left plot of group (a 'group' is an analysis of identical markers).
- [x] All plots are contour plots with outliers or pseudocolor plots.
- [x] A numerical value for number of cells or percentage (with statistics) is provided.

Methodology

Sample preparation	General FACs immunostaining.<br>Lungs, kidney, spleen, and mediastinal lymph node tissues were harvested, and lymphocytes isolated. Next, lymphocytes were stained in vitro with anti-CD8α antibodies along with fluorochrome conjugated antibodies specific of other surface markers resuspended in FACS buffer (PBS/1% fetal bovine serum). For immunostaining of intracellular markers such as transcription factors T-bet, Eomes or Nur77, after staining of cell surface markers, cell samples were rinsed, fixed and permeabilized with Cytofix/cytoperm. Stained cells were run on a LSR Fortessa flow cytometer (BD, San Jose, CA, data collected with FACS Diva 6.0 by BD) or run on a Cytek Aurora spectral flow cytometer using the Cytek Spectraflo 3.0.3 software. Flow cytometry data was analyzed using with FlowJo software version 10.4 (Tree Star, Inc., Ashland, OR). It should be considered that our observations refer mainly to the generation and maintenance of influenza specific memory CD8 T cells in the lung parenchyma identified through IV labeling, a method widely used to study TRM cells in the field. One limitation of this approach is that it can only provide a snapshot of the cells that are residents in tissue at a given time and it is less accurate at quantifying T cells in transition. A table of antibodies is provided in supplementary information.

Intracellular cytokine staining. Lymphocytes were isolated from the lungs of VSV-OVA-challenged mice and stimulated ex vivo with OVA peptide 1M) in the presence of Golgi-Plug (BD Biosciences) for 5 hours. Following incubation, cells were harvested and antigen specific CD8+ T cells were assessed for the expression of TNF and IFNg by flow cytometry as in ref. 44.

Intracellular phosphorylated-SMAD2/3 and phosphorylated-NFkB detection by immunostaining for flow cytometry. Cell suspensions from lung, lymph nodes or spleen were fixed with 4% Formaldehyde aqueous solution for 20 min at room temperature, followed by two rinses with PBS/1% fetal bovine serum solution (FACs buffer). Permeabilization was performed with ice cold methanol at -20oC overnight. The next day, cells were stained with anti-phospho SMAD2/3 or anti-phospho NFkB in PBS/1% fetal bovine serum solution. For Phospho NFkB staining, inhibitors of phosphatases (sodium pervanadate 1mM, and sodium fluoride 100mM) were added at the same time.

Instrument	LSRII Fortessa (BD) , Aurora (Cytek) spectral flow cytometers.
Software	FACS Diva 6.0 ,Cytek Spectraflo 3.0.3 software. FlowJo FACS analysis software (Tree Star, Inc.) version 10.4
Cell population abundance	In experiments using CD8+ T cell enrichment (magnetically sorted cells), all donor cells were assessed by flow cytometry to verify 90-95% purity and/or frequency of CD8+ T cells.
Gating strategy	Indicated in Figure lends and supplementary information. In brief, live lymphocytes were identified and debris was excluded using FSC x SSC. Single cells were then identified using FSC-H x FSC-A and cells not lying along the 45 degree boundary were excluded. Non-stained and cell samples from non-infected mice were utilized to identify negative populations when relevant. Appropriate isotype controls were used to identify and subtract background fluorescence in experiments in which MFI levels were measured.

☒ Tick this box to confirm that a figure exemplifying the gating strategy is provided in the Supplementary Information.
